# Effects of all-trans retinoic acid on glutamate receptor expression and dendritic spine density in the hypothalamus of rats

**DOI:** 10.3389/fnins.2025.1616330

**Published:** 2025-08-18

**Authors:** Yuan-Zhong Kai, Chun Xia, Qing-Hong Shan, Zheng-Quan Tang

**Affiliations:** ^1^The School of Life Sciences, Anhui University, Hefei, Anhui, China; ^2^Qilin Middle School, Nanshan Experimental Education Group, Shenzhen, Guangdong, China; ^3^Institute of Brain Science, The First Affiliated Hospital of Anhui Medical University, Hefei, Anhui, China

**Keywords:** all-trans retinoic acid, glutamate receptor, dendritic spine, hypothalamus, depression, rat

## Abstract

Increasing evidence suggests that all-trans retinoic acid (ATRA) contributes to the pathogenesis of depression. Although numerous studies have emphasized the role of hippocampal glutamatergic transmission and dendritic spine abnormalities in ATRA-induced depression, it remains unclear whether changes in hypothalamic glutamatergic transmission and dendritic spine density also contribute to its development. This study investigates the effects of ATRA treatment on glutamate receptor expression and dendritic spine density in the hypothalamus of rats. We found that prolonged ATRA exposure induced depression-like behaviors, as evidenced by altered behavior in standard tests. These behavioral changes were accompanied by significant upregulation of retinoic acid receptor *α* (RARα) and corticotropin-releasing hormone (CRH) expression in the hypothalamus, suggesting a potential involvement of retinoic acid signaling in the regulation of stress-related pathways. Furthermore, rats exhibiting depression-like behaviors after ATRA treatment showed abnormal expression patterns of AMPA receptor subunits. ATRA treatment significantly increases dendritic spine density in hypothalamic neurons, particularly in secondary and tertiary dendrites. Most importantly, significant increases were observed in the GluR1, GluR2, and GluR4 subunits of the AMPA receptor, along with a slight increase in primary dendrite numbers. Additionally, there were significant increases in spine density on secondary and tertiary dendrites, which are critical for synaptic plasticity and neurotransmission. These findings point to a potential disruption in glutamatergic signaling in response to chronic ATRA exposure. In parallel, we observed a significant increase in dendritic spine density in cultured hypothalamic neurons following ATRA treatment. This effect was most pronounced in secondary and tertiary dendrites, indicating a selective enhancement of dendritic complexity. These results suggest that ATRA may alter both synaptic structure and glutamatergic function in the hypothalamus, providing new insights into its potential role in stress-related disorders. Our study highlights the importance of retinoic acid signaling in modulating neural plasticity and provides a novel perspective on the molecular mechanisms underlying ATRA-induced mood disturbances.

## 1 Introduction

Retinoic signaling, particularly through vitamin A derivatives, has been implicated in the pathogenesis of depression (Hu et al., 2020). Excessive vitamin A intake (hypervitaminosis A) and synthetic retinoids like 13-cis-retinoic acid (Accutane) have been associated with psychiatric symptoms, including depression and mood disturbances (Hull and D’Arcy, 2003; Shalita et al., 1988). Chronic use of these compounds can lead to depression-like behaviors in both humans and animal models (Hu et al., 2020; Restak, 1972). All-trans retinoic acid (ATRA), the biologically active metabolite of vitamin A, plays a pivotal role in numerous physiological processes, including neural development, synaptic plasticity, and gene transcription (Jacobs et al., 2006; Werner and Deluca, 2002). Recent studies suggest that ATRA contributes to depression through multiple mechanisms (Yang et al., 2020). ATRA has been shown to suppress hippocampal neurogenesis, a process critical for mood regulation and cognitive function (Hu et al., 2016). Furthermore, ATRA appears to dysregulate the expression of synaptic genes in hippocampus, potentially disrupting synaptic plasticity and neurotransmission (Chen et al., 2009; Qin et al., 2020). These studies collectively highlight ATRA’s role in the pathogenesis of depression by altering neurobiological pathways involved in stress responses and synaptic function in hippocampus (Aoto et al., 2008).

The hypothalamus, a critical region of the brain, plays a significant role in regulating emotional responses, stress, and physiological processes. Research suggests that dysfunction in the hypothalamus, particularly its interaction with the hypothalamic–pituitary–adrenal (HPA) axis, may contribute to the development or exacerbation of depressive symptoms. The HPA axis controls the body’s stress response by releasing hormones like cortisol, and chronic overactivation of this system is often observed in individuals with depression. Recent studies suggest that ATRA-induced activation of the hypothalamus-pituitary–adrenal (HPA) axis is a crucial aspect of the pathophysiology underlying neuropsychiatric symptoms such as depression and anxiety. The impairments in glucocorticoid receptor (GR) negative feedback and the increased expression of retinoic acid receptor alpha (RAR-*α*) in the hypothalamus are central mechanisms involved in the dysregulation of the hypothalamus-pituitary–adrenal (HPA) axis and the resulting stress and depression-like behaviors (Hu et al., 2013). Long-term ATRA administration in young rats has been shown to induce HPA axis hyperactivity and behavioral changes, suggesting a potential link to depressive-like states (Cai et al., 2010). However, the precise mechanisms underlying ATRA-induced hypothalamic dysregulation and its role in depression remain unclear.

Depression is a complex neuropsychiatric disorder with multifaceted pathophysiology. Emerging evidence suggests that alterations in AMPA receptor function and dendritic spine abnormalities play significant roles in the development of depression (Barbon et al., 2011; Qiao et al., 2016). AMPA receptors, which mediate fast excitatory neurotransmission, are implicated in synaptic plasticity and mood regulation. Chronic stress, a common risk factor for depression, disrupts AMPA receptor function and expression, leading to impaired synaptic transmission in brain regions such as the hippocampus, amygdala, and prefrontal cortex (Lee et al., 2022). These changes are associated with reduced dendritic spine density and complexity, particularly in the hippocampus, where chronic stress reduces neurogenesis and synaptic connectivity (Qiao et al., 2016). Additionally, chronic stress alters AMPA receptor subunit composition, enhancing excitatory transmission in the amygdala and contributing to emotional dysregulation. Dendritic spine abnormalities further exacerbate synaptic dysfunction, weakening inhibitory signaling to the HPA axis and perpetuating stress responses (Qiao et al., 2016). Therefore, to underscore the importance of AMPA receptors and dendritic spine integrity in the pathophysiology of depression and highlight potential avenues for developing new, more effective antidepressant therapies.

The objective of our study is to explore how ATRA treatment influences AMPA receptor subunit expression and dendritic spine density in the hypothalamus, particularly in the context of depression-like behaviors in young rats. This research aims to provide insights into the potential neurobiological mechanisms underlying ATRA’s effects on hypothalamic function and its implications for depression.

## 2 Experimental procedures

### 2.1 Animals

Male Sprague–Dawley rats (SHANGHAI SLAC LABORATORY ANIMAL CO., LTD, Shanghai, China) were 3 weeks old upon arrival and 4 weeks old at the start of treatment. The rats were housed in colony cages with food and water provided ad libitum, and maintained on a 12 h light–dark cycle (lights on at 8:00 AM). The ambient temperature was kept at 21–22°C with 50–60% relative humidity. The animals were allowed to acclimate for 1 week, during which they were handled daily. Upon arrival, the rats were weighed, and subsequent measurements were taken weekly throughout the experiment. All animal procedures were approved by the Institutional Animal Care and Use Committee (IACUC) of Anhui University.

### 2.2 Animal treatment

All animals received daily intraperitoneal injections at a volume of 1 mL/kg body weight. The vehicle control group (*n* = 15) was administered a sterile saline solution (0.9% w/v sodium chloride) with dimethyl sulfoxide (DMSO) in a 1:1 v/v ratio (Trent et al., 2009). The drug-treated group (*n* = 15) received 2 mg/kg ATRA (Sigma, St. Louis, MO) dissolved in a 1:1 v/v mixture of DMSO and saline. ATRA solutions were prepared under red light, and the flasks containing ATRA were wrapped in aluminum foil to prevent photoisomerization (O’Reilly et al., 2006). All animals were treated for 6 weeks before the behavioral tests began, and the treatments continued throughout the duration of the tests. To minimize any acute effects of the injections on behavior, injections were administered between 17:00 and 18:00, while behavioral tests were conducted between 10:00 and 14:00. Only one behavioral test was performed per day, with approximately 24 h between each test. The tests were carried out in the following order: sucrose preference test, open-field test, elevated plus maze (EPM), tail-suspension test (TST), and forced swimming test (FST). Rats were tested in random order for each behavioral test, and all behaviors were scored by a trained observer who was blinded to the treatment conditions. Daily monitoring confirmed that the animals did not exhibit signs of distress from the repeated injections, which were alternately administered on both sides of the abdominal cavity.

### 2.3 Behavioral tests

#### 2.3.1 Sucrose-preference test

Sucrose intake is commonly used as a measure of anhedonia, a core symptom of depression. In the sucrose preference test, all animals were housed individually and initially trained to drink a 2% (w/v) sucrose solution for 24 h. Following a 12-h period of food and water deprivation, the animals were provided with free access to two bottles—one containing normal water and the other a 2% sucrose solution. The bottles were weighed at the beginning and end of the 24-h testing period to assess the amount of sucrose and water consumed.

#### 2.3.2 Open-field test

The open-field apparatus was used to assess the animals’ spontaneous exploratory activity and curiosity in a novel environment, as well as to evaluate anxiety-related behaviors (Chen et al., 2009; Mathis et al., 1994; Prut and Belzung, 2003; Ramos and Mormède, 1998). The open-field apparatus was made of black wood and consisted of a 100 cm × 100 cm square arena with 30 cm high black walls. The floor was marked with 6-mm white lines to divide the area into 16 equal squares, with the four central squares designated as the center area. The test was conducted under bright ambient room lighting. Each rat was placed in the center of the arena and allowed to explore the unfamiliar environment for 5 min (Cai et al., 2010).

#### 2.3.3 Elevated plus maze

The elevated plus maze (EPM) is commonly used alongside the open-field test to assess exploratory behavior and anxiety-related responses in animals (Cai et al., 2010; Walf and Frye, 2007). The EPM was made of black Plexiglas and comprised two opposite open arms (50 cm × 10 cm × 0.5 cm), an open central platform (10 cm × 10 cm), and two opposite enclosed arms (50 cm × 10 cm × 40 cm). The maze was elevated 50 cm above the floor and illuminated with dim overhead lighting. Each rat was placed on the central platform, facing one of the open arms, and allowed to explore the apparatus for 5 min (Cai et al., 2010). The time spent in the open arms and the frequency of entries into the open arms were recorded and analyzed using a video camera. To prevent the odor of previous subjects from influencing the behavior of the next rat, the arms were wiped with 75% alcohol at the end of each trial.

#### 2.3.4 Tail-suspension test

The TST was conducted according to the method of Yamawaki et al., with a slight modification (Yamawaki et al., 2012). Briefly, the rats were suspended by their forelimbs using bands and hung from a mounted hook 50 cm above the floor for a 5-min test period. The session was videotaped for later analysis, and the time spent immobile was recorded. Immobility was defined as the absence of all movement, except for whisker movements and respiration.

#### 2.3.5 Forced swimming test

The FST was conducted according to the method described in our previous study (Zhu et al., 2009). The behavioral cylinder was 60 cm high and 25 cm in diameter, maintained at 24–25°C, and filled with 50 cm of water so that the rats could not support themselves by touching the bottom with their paws or tail. The FST paradigm included two sections: an initial 15-min pretest, followed by a 5-min test 24 h later. After each session, the rats were removed from the cylinders, dried with towels, placed into heated cages for 15 min, and then returned to their home cages. The rats were considered immobile when they did not make any active movements. The rats were considered to be struggling when they made active movements with their forepaws in and out of the water along the side of the swim chamber. The rats were considered to be swimming when they were actively moving their forepaws in circular movements.

### 2.4 Tissue preparation

After the last behavioral test, the rats were transferred to the laboratory for decapitation. The rats were anesthetized and decapitated between 09:00 and 14:00, and the members of the two groups were sacrificed randomly. Whole brains were collected immediately. The whole brains of the 14 rats in the ATRA-treated group and those of the 15 rats in the vehicle group were randomly assigned to the subsequent measurements (Cai et al., 2010). Hypothalamic tissues were dissected from the brains with the following boundaries: anterior border of the optic chiasm, anterior border of the mammillary bodies, and lateral hypothalamic sulci. The depth of dissection was approximately 3 mm. The tissues were quickly frozen in liquid nitrogen and stored at −80°C (Bliss and Collingridge, 1993; Huang et al., 2008).

### 2.5 Co-localization of glutamate receptors and RAR-*α* in hypothalamic CRH neurons

The rats were euthanized with an intraperitoneal injection of sodium pentobarbital and decapitated; the brain was quickly removed from the skull and frozen in liquid nitrogen prior to storage at −80°C. For cryosectioning, the frozen rat brain was transferred from −80°C to the cryostat chamber at −20°C and then sectioned at 10 μm. Sections containing the hypothalamic paraventricular nucleus (PVN) were collected, mounted on a slide, and then fixed with 4% PFA for 10 min. For triple-labeling immunofluorescence, the sections were permeabilized with PBS containing 0.3% Triton X-100 for 20 min. After blocking in 5% donkey serum for 30 min, the sections were simultaneously incubated with three primary antibodies overnight at 4°C, washed, and then simultaneously incubated with three secondary antibodies at room temperature for 2 h. After washing, the sections were mounted with 80% glycerol, and the images were captured with a Zeiss710 confocal laser scanning microscope. The following primary antibodies were used: guinea pig polyclonal anti-CRF antibody (1:2,000, t5007, San Carlos, California, United States), mouse monoclonal anti-glutamate receptor R1 antibody (1:100, SC55509, Santa Cruz Biotechnology, United States), and rabbit polyclonal anti-RAR-*α* antibody (1:100, SC551, Santa Cruz Biotechnology, United States). The secondary antibodies were Alexa Fluor-488 AffiniPure donkey anti-mouse antibody (1:200, Jackson ImmunoResearch), Alexa Fluor-594 AffiniPure donkey anti-guinea pig antibody (1:200, Jackson ImmunoResearch), and Dylight-405 AffiniPure donkey anti-rabbit antibody (1:200, Jackson ImmunoResearch).

### 2.6 UPLC system for the determination of glutamate concentration

The hypothalamic tissues were homogenized in 15 vol. of methanol/water (85:15, v/v), centrifuged (8,000 × g for 15 min at 4°C), and aliquots of the supernatants were stored at −20°C until derivatization for the glutamate analysis. The UPLC system consisted of a Shimadzu chromatograph equipped with a 3-μm particle size (150 mm × 4.6 mm, ID) C18 analytical column (Hibar-Futigsanle RT). An integrator (Shimadzu C-R7Ae plus) was used to analyze the chromatographic data. HPLC grade methanol was obtained from J. T. Baker (Phillipsburg, NJ, United States). The sodium acetate, tetrahydrofuran, acetic acid, OPA, 3-mercaptopropionic acid (MPA) and amino acid standards were obtained from Sigma-Aldrich (St. Louis, MO, United States). All of the other reagents were of the highest purity available, and the water was Milli-Q deionized water. The procedure used here has been described previously (Clarke et al., 2007; de Freitas Silva et al., 2009). Briefly, the samples were derivatized by mixing 100 μL of the sample or standard solutions with 20 μL of freshly prepared methanolic OPA (5 mg/mL), 75 μL borate buffer (pH 9.9) and 5 μL MPA. The resulting solution was vortexed and analyzed after 1 min of incubation at room temperature in the dark. The mobile phase consisted of 0.05 M sodium acetate, tetrahydrofuran, and methanol (50:1:49, v/v) adjusted to pH 4.0. The mobile phase was filtered through Millipore 0.45-μm Durapore membrane filters and vacuum degassed prior to use. The chromatographic analyzes were performed at 25 ± 2°C. The compounds were eluted isocratically over a 5 min runtime at a flow rate of 1 mL/min. The fluorescent detector was set at an excitation wavelength of 337 nm, an emission wavelength of 454 nm, a low sensitivity, and range of 2×. The obtained values are reported as μg/g of tissue.

### 2.7 RNA isolation and real-time quantitative polymerase chain reaction

Total RNA was extracted from the frozen hypothalamus using the Trizol (Invitrogen, Carlsbad, CA) method. cDNA was synthesized using reverse transcriptase (Promega, Wisconsin, United States). qPCR was performed using a SYBR Green PCR Kit (Applied Biosystems, United States) and an ABI Prism 7000 Sequence Detector system in a 25-μl volume for 40 cycles (15 s at 95°C; 60 s at 64°C for rat CRH and RAR-*α*). The following primers were used: rat CRH 5′-CAGAACAACAGTGCGGGCTCA-3′ and 5′-AAGGCAGACAGGGCGACAGAG-3′; rat RAR-α 5′-ACCATTGCCGACCAGATTACCC-3′ and 5′-AAGGTCATTGTGTCTTGCTCAGGT-3′; AMPA R1 5′GAGCAACGAAAGCCCTGTGA-3′ and 5′CCCTTGGGTGTCGCAATG-3′; AMPA R2 5′AACGAGTACATCGAGCAGAGGAA-3′ and 5′GATGCCGTAGCCTTTGGAATC3′; AMPA R3 5′TTCGGAAGTCCAAGGGAAAGT-3′ and 5′CACGGCTTTCTCTGCTCAATG3′; AMPA R4 5′GGCTCGTGTCCGCAAGTC-3′ and 5′TTCGCTGCTCAATGTATTCATTC3′; and rat beta-actin 5′-TTGCTGACAGGATGCAGAA-3′ and 5′-ACCAATCCACACAGAGTACTT-3′. The relative amount of the target mRNA was calculated using the 2^−ΔΔCt^ method (Livak and Schmittgen, 2001). The relative amplification efficiencies of the primers were tested and shown to be similar.

### 2.8 Western blot analysis

The levels of AMPA receptor and PSD95 in the hypothalamus were assessed via Western blotting. Protein concentration was quantified using a BCA assay (Bio-Rad Laboratories, Hercules, CA). Immunoblots for AMPA receptor, RAR-*α*, synapsin I, and PSD95 were performed using the following primary antibodies: rabbit anti-rat AMPA receptor (1:1,000, mouse monoclonal IgG for GluR1, sc-55509), rabbit polyclonal IgG for RAR-α (1:500, sc-551), rabbit polyclonal IgG for synapsin I (1:500, AB1543P), and rabbit polyclonal IgG for PSD95 (1:500, 610,496). The intensities of the bands corresponding to AMPA receptor, RAR-α, and PSD95 were normalized to the corresponding GAPDH (*in vitro*) signal. Band detection was performed using the Rodeo™ ECL Western Blotting Detection Kit (enhanced chemiluminescence, 72,550, USB Corporation, Cleveland, OH, United States). Relative density measurements were used for quantification, with the ratios of the AMPA receptor, RAR-α, and PSD95 band intensities to the intensity of the internal control (GAPDH) bands providing estimates of the respective protein concentrations.

### 2.9 Primary culture of E18 hypothalamic neurons from SD rats

Pregnant SD rats were deeply anesthetized by carbon dioxide gas (CO2) inhalation, followed by abdominal disinfection and uterine extraction. E18 fetal rats were isolated and placed in HBSS. Brains are extracted by decapitation, and the hypothalamus is identified under a stereomicroscope using anatomical landmarks between the tuber cinereum and optic chiasm. The hypothalamus is divided into four regions (preoptic, supraoptic, tuberal, and mamillary) and three zones (periventricular, medial, and lateral), with key nuclei including the supraoptic, paraventricular, and ventromedial nuclei. Tissue is dissected into ~2 mm^2^ pieces, digested in 0.25% trypsin/0.02% EDTA at 37°C until edges appear translucent, and triturated to form a cell suspension. Cells are counted using a hemocytometer, adjusted to 2–5 × 10^5^ cells/ml, and plated in DMEM/F-12 medium containing 10% FBS and antibiotics. Cultures are incubated at 37°C with 5% CO₂. After 4 h, cells are washed with pre-warmed serum-free medium to remove debris and fed with NEUROBASAL-A medium supplemented with 2% B27, antibiotics, and 2 mM glutamine. Medium is refreshed every 3–4 days. If glial overgrowth is observed on days 5–6, cytosine arabinoside (AraC) is added. Incubator conditions (temperature, CO₂ levels, and humidity) are monitored regularly to ensure optimal growth. Glassware is coated with poly-L-lysine, rinsed with sterile ddH₂O, and pre-warmed with medium before use.

### 2.10 Dynamic imaging of hypothalamic neurons

To prepare glass chamber slides for live-cell imaging, coat with 0.05% poly-L-lysine and allow to settle overnight. Rinse thoroughly with sterilized ddH₂O, aspirate, and air-dry in a sterile environment. Pre-warm the culture medium and prepare primary hypothalamic neuron cultures from E18 Sprague–Dawley rats following established protocols. After 2 weeks of culture, when neurons are mature, remove plates from the incubator and add LIVE/DEAD Viability/Cytotoxicity dye (Invitrogen) to the medium in a light-protected environment. Incubate at 37°C for 30 min, then aspirate the medium and wash cells three times with PBS, keeping all steps shielded from light. Replace PBS with pre-warmed Neurobasal medium supplemented with 2% B27, 1‰ penicillin–streptomycin, and 2 mM glutamine. Perform live-cell confocal imaging of dendritic spine dynamics using an LSM710 laser scanning confocal microscope, capturing neuronal activity over 30 min. Add all-trans retinoic acid (ATRA; 1 μM in DMSO) under light-protected conditions and continue dynamic imaging for an additional 30 min. After treatment, aspirate the ATRA-containing medium, replace with fresh pre-warmed supplemented Neurobasal medium, and continue live imaging for 1 h to monitor post-treatment spine dynamics.

### 2.11 Statistical analyzes

All statistical analyzes were performed using GraphPad Prism for Windows. Data are presented as means ± standard errors of the mean (SEM). The differences between the vehicle and ATRA groups were assessed using independent-samples *t*-tests with 95% confidence intervals. *p* < 0.05 was considered statistically significant.

## 3 Results

### 3.1 Chronic administration of ATRA resulted in depression-like behaviors in rats

**Figure 1:**
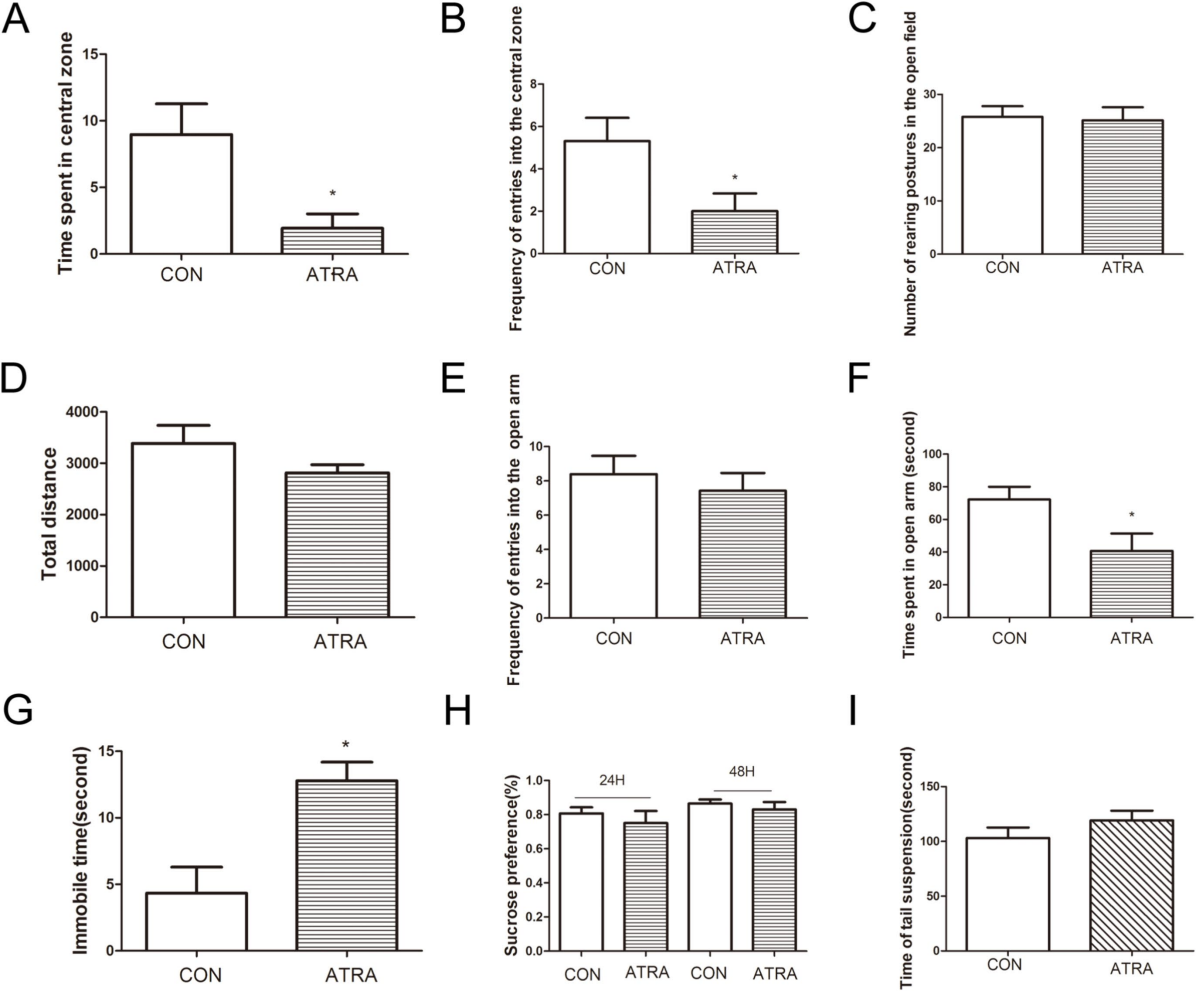
ATRA administration induced depression-like behavior in rats. **(A,B)** The ATRA group exhibited significantly reduced time spent in the central zone (*p* < 0.05) and fewer entries into the central zone (*p* < 0.05) compared to the control group. **(C,D)** No significant differences were observed between groups in rearing postures or total distance traveled during the open-field test. **(E,F)** In the elevated plus maze (EPM) test, the ATRA group spent less time in the open arms (*p* < 0.05) and had fewer entries into the open arms (*p* < 0.05) compared to the control group. **(G)** During the forced swim test (FST), the ATRA group showed significantly increased immobility time (*p* < 0.05). **(H)** Specifically, immobility time in the ATRA group was 12.79 ± 1.39 s versus 4.33 ± 1.96 s in the control group (*p* < 0.05). **(I)** In the tail-suspension test (TST), immobility times were 119.1 ± 8.91 s (ATRA) and 103.0 ± 9.74 s (control), with no significant difference between groups. Additionally, no significant differences were observed between the ATRA and control groups in the sucrose-preference test at 24 h or 48 h. Data are presented as mean ± SEM. Student’s *t*-tests were used for statistical analysis. Sample sizes were *n* = 15 per group (CON and ATRA). Statistical significance (*p* < 0.05) is denoted by asterisks (*) in the figure.

In the current study, we investigated whether chronic ATRA administration induces depression-like behaviors in rats using a battery of behavioral tests. In the open-field test, the ATRA-treated group exhibited reduced central zone exploration, with significantly less time spent (Figure 1A, *p* < 0.05) and fewer entries (Figure 1B, *p* < 0.05) compared to controls. No group differences were observed in rearing postures (Figure 1C, *p* > 0.05) or total distance traveled (Figure 1D, *p* > 0.05). In the elevated plus maze (EPM), the ATRA group showed anxiety-like behavior, with fewer entries into (Figure 1E) and less time spent in the open arms (Figure 1F, *p* < 0.05). In the forced swim test (FST), ATRA-treated rats displayed increased immobility time (Figure 1G, *p* < 0.05; 12.79 ± 1.39 s vs. 4.33 ± 1.96 s in controls). No significant differences emerged in the sucrose preference test (Figure 1H, *p* > 0.05) at 24 or 48 h, or in the tail-suspension test (TST; Figure 1I, *p* > 0.05; 119.1 ± 8.91 s vs. 103.0 ± 9.74 s). These findings indicate that chronic ATRA administration is associated with depression-like behaviors in rats.

### 3.2 Effects of ATRA treatment on the hypothalamic glutamate system

**Figure 2:**
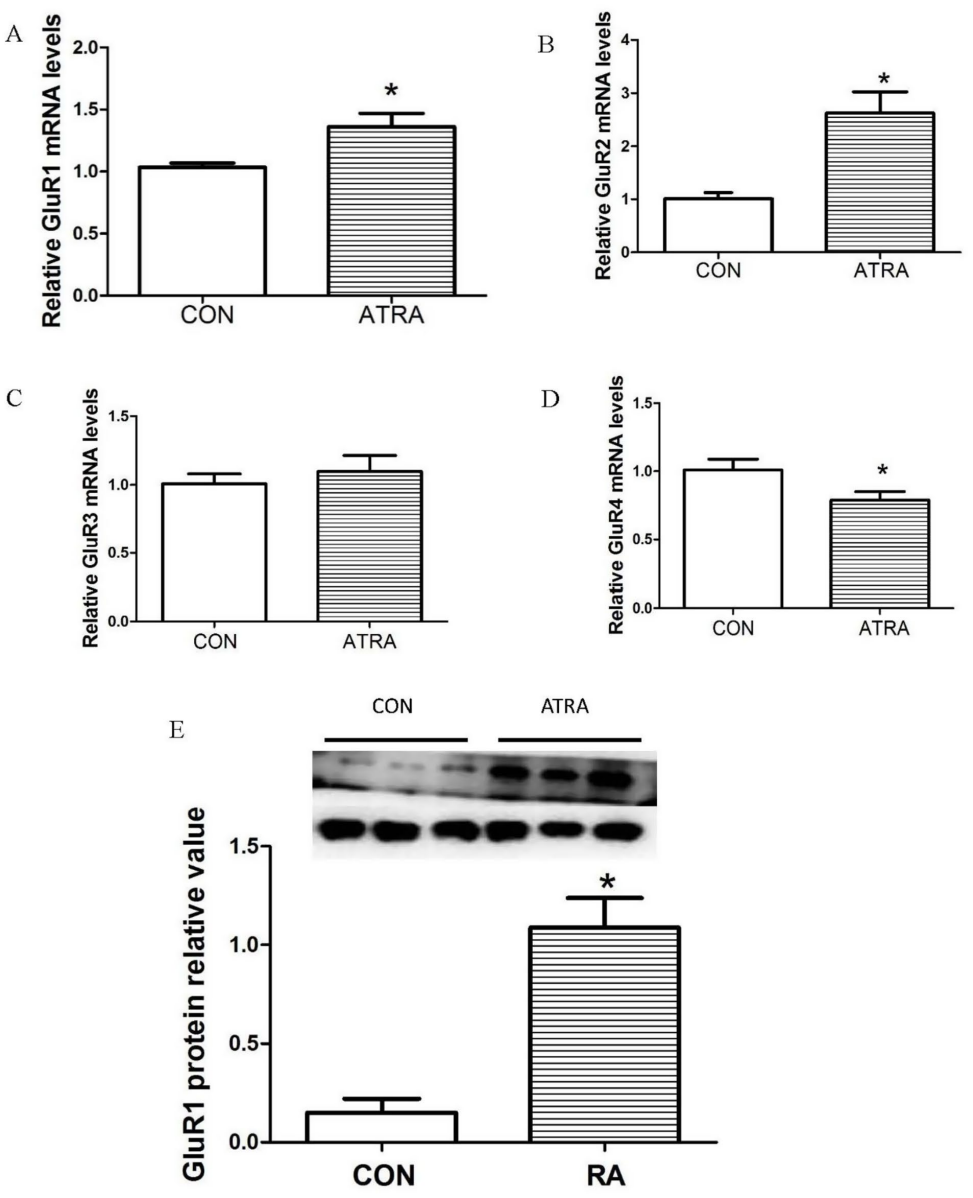
Effects of chronic ATRA administration on the expression of AMPA receptor subunit mRNAs and proteins. **(A,B)** Hypothalamic mRNA expression levels of GluR1 and GluR2 were significantly elevated in the chronic ATRA-treated group compared to the control group. **(C,D)** GluR3 mRNA expression showed no significant change in the ATRA-treated group relative to the control group, whereas GluR4 mRNA expression was significantly reduced in the ATRA-treated group. **(E)** GluR1 protein levels were significantly increased in the ATRA-treated group compared with the control group. Data are presented as mean ± SEM; Student’s *t*-tests were used for analysis. Statistical significance (*p* < 0.05) is denoted by asterisks (*) in the figure.

To investigate the impact of ATRA treatment on the rat glutamate system, we measured the mRNA expression levels of GluR1, GluR2, GluR3, and GluR4 in the hypothalamus using real-time qPCR. Results showed that chronic ATRA treatment significantly increased GluR1 (Figure 2A, *p* = 0.023) and GluR2 (Figure 2B, *p* = 0.021) mRNA expression compared to vehicle-treated controls. No significant differences were observed in GluR3 mRNA levels between groups (Figure 2C, *p* = 0.268). Notably, GluR4 mRNA expression was significantly reduced in the ATRA-treated group (Figure 2D, *p* = 0.036). Western blot analysis further revealed a significant upregulation of GluR1 protein levels in the ATRA-treated group (Figure 2E, *p* = 0.038).

### 3.3 ATRA disrupted synaptic homeostasis

**Figure 3:**
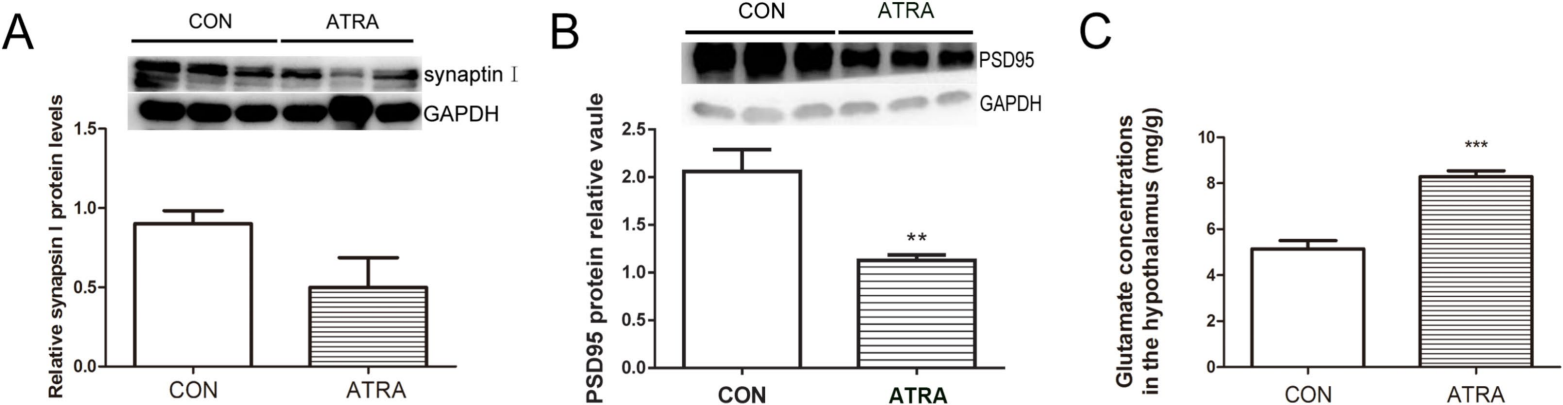
ATRA disrupts synaptic homeostasis by altering the expression of synaptic proteins. **(A,B)** No significant differences in synapsin I levels were observed between the two groups. However, PSD95 protein levels were significantly reduced in the ATRA-treated group. **(C)** Glutamate concentrations in the homogenized hypothalamus, measured using UPLC and expressed as mg/g of tissue, were significantly elevated in the ATRA-treated group. Data are presented as mean ± SEM; Student’s *t*-tests were used for analysis. Statistical significance between the CON and ATRA groups is denoted by asterisks (**, *p* < 0.05; ***, *p* < 0.01) in the figure.

To evaluate whether ATRA-induced imbalances in synaptic protein expression occur at the synaptic level, synapsin I and PSD-95 protein levels were assessed via Western blotting, and glutamate concentrations were measured using UPLC. No significant changes were observed in synapsin I protein levels between groups (Figure 3A, *p* = 0.121). However, PSD-95 protein levels were significantly reduced in the ATRA-treated group (Figure 3B, *p* = 0.003). UPLC quantification of hypothalamic glutamate concentrations, expressed as mg/g of tissue, revealed a significant increase in the ATRA-treated group compared to controls (Figure 3C, *p* = 0.007). These findings suggest that ATRA disrupts synaptic homeostasis in the hypothalamus.

### 3.4 ATRA upregulated CRH and RARα expression in the hypothalamus

**Figure 4:**
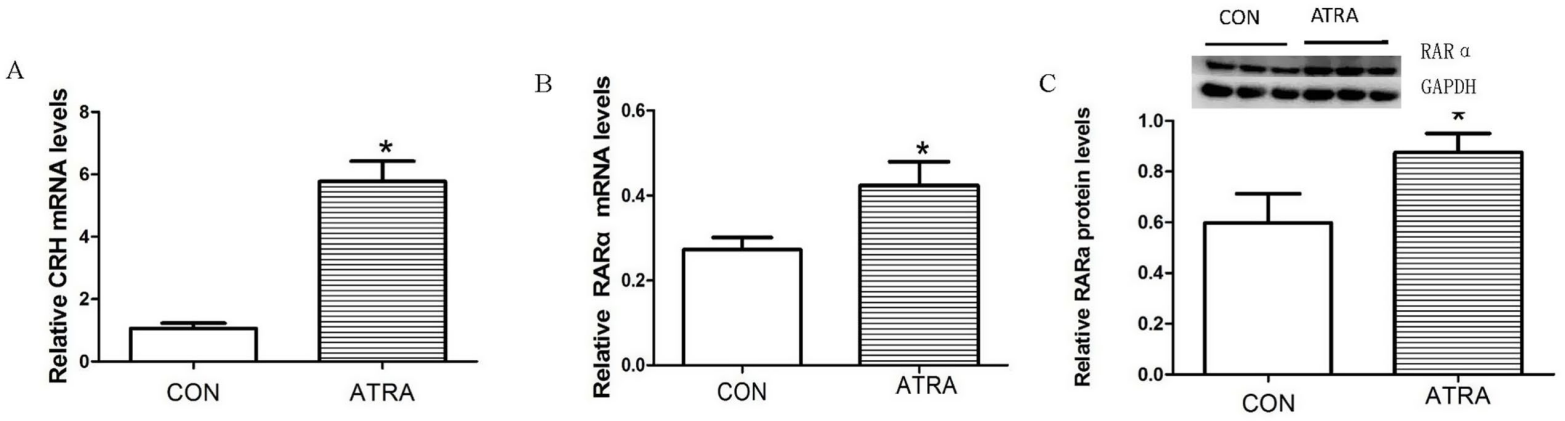
The effects of chronic ATRA administration on RAR-*α* and CRH expression. **(A,B)** CRH and RAR-α mRNA expression levels were significantly increased in the ATRA-treated group versus the control group. **(C)** RAR-α protein levels were significantly increased in the ATRA-treated group compared with the control group. Data are presented as mean ± SEM and analyzed using Student’s *t*-tests. Statistical significance (*p* < 0.05) is denoted by asterisks (*) in the figure.

Real-time qPCR analysis showed that RARα and CRH mRNA levels were significantly elevated in the hypothalamus of ATRA-treated rats compared to vehicle-treated controls (Figure 4A, *p* = 0.002; Figure 4B, *p* = 0.043). Consistently, Western blotting confirmed a significant increase in RARα protein levels in the ATRA group (Figure 4C, *p* = 0.025). These results demonstrate that ATRA treatment upregulated both CRH and RARα expression in the hypothalamus.

### 3.5 Co-localization of RARα and GluR1 in CRH-expressing hypothalamic neurons

**Figure 5:**
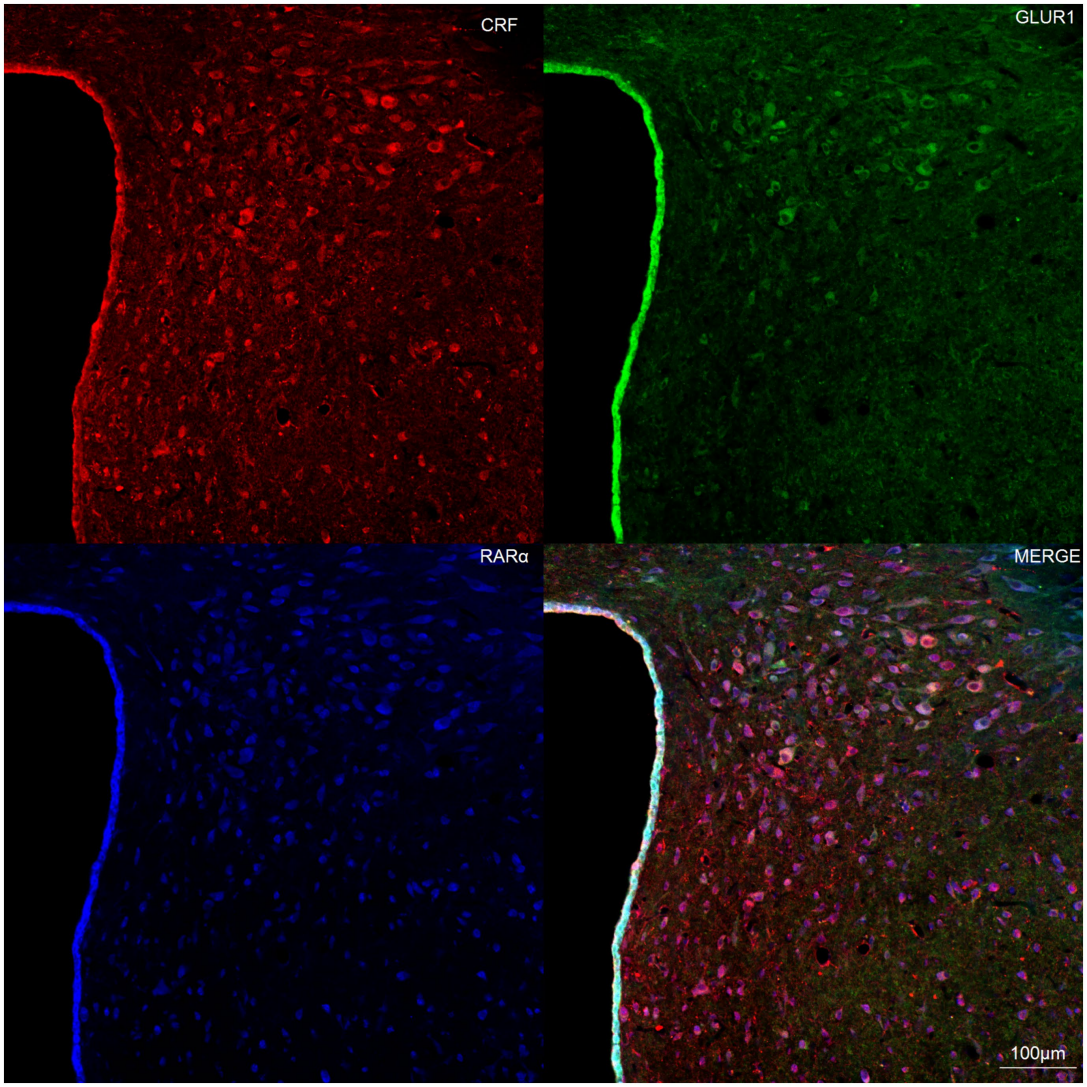
Representative laser confocal immunofluorescence image of hypothalamic neurons. Brain sections (10 μm thick) from the hypothalamic paraventricular nucleus (PVN) were mounted onto slides. Immunofluorescence staining demonstrated co-localization of RAR-α (blue) and GluR1 (green) in CRH-positive neurons (red). Scale bar = 100 μm.

To investigate the relationship between RARα and glutamate receptors in CRH-expressing neurons of the hypothalamic paraventricular nucleus (PVN), immunohistochemistry was performed on rat brain slices. Results revealed co-expression of RARα and GluR1 in CRH-positive neurons within the hypothalamus (Figure 5). These findings suggest that many neurons in the hypothalamic CRH population co-express both RARα and GluR1.

### 3.6 ATRA modulated the correlation between GluR1, CRH, and RARα mRNA levels

**Figure 6:**
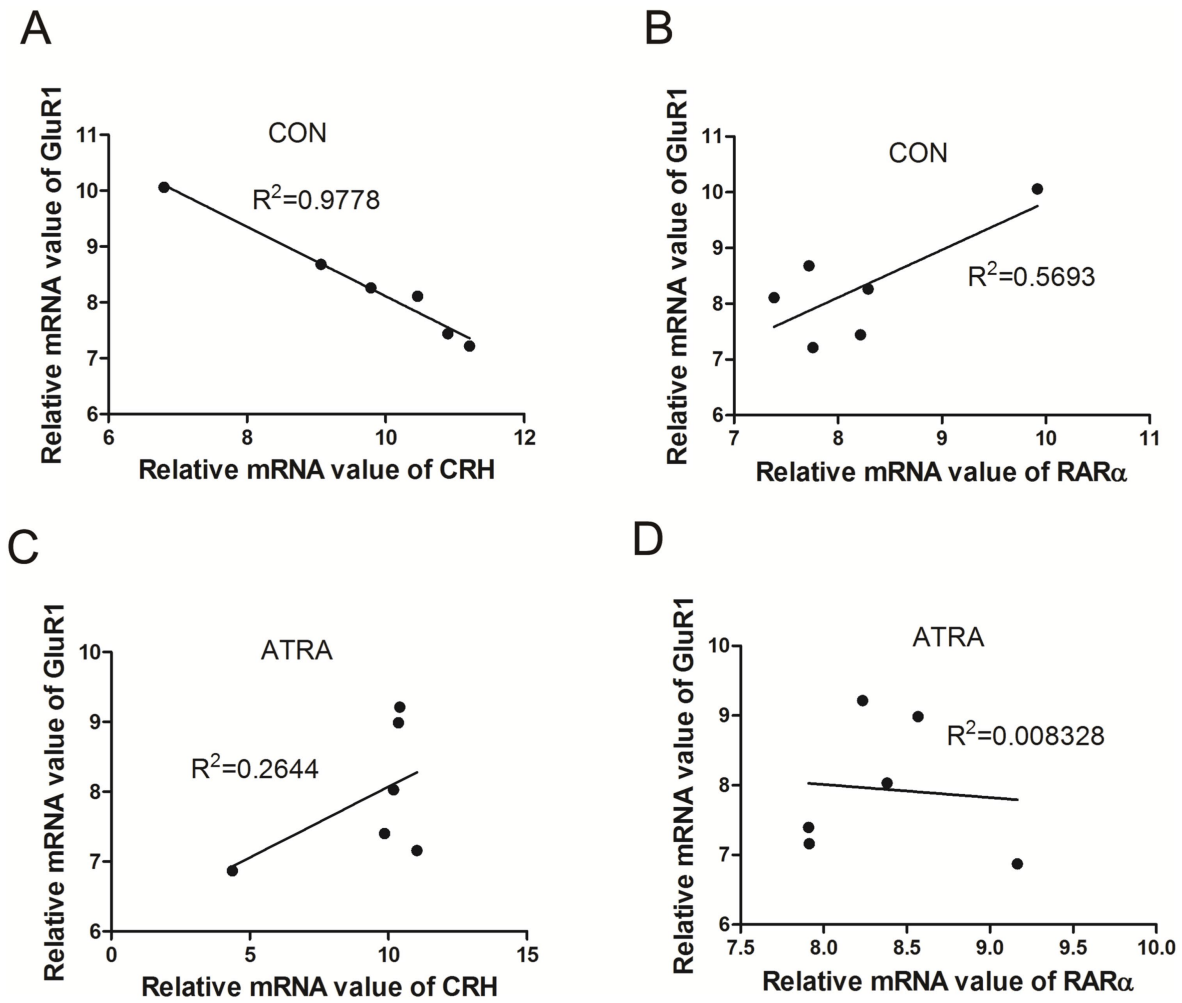
Correlations between GluR1, CRH, and RAR-α mRNA levels. **(A,B)** In the control group, GluR1 and CRH mRNA levels showed a significant negative correlation (*r* = −0.989, **p = 0.0001, **A**), while GluR1 and RAR-α mRNA levels exhibited a positive correlation (*r* = 0.755, *p* = 0.083, **B**). **(C,D)** In the ATRA-treated group, GluR1 and CRH mRNA levels demonstrated a positive correlation (*r* = 0.514, *p* = 0.297, **C**), whereas no significant correlation was observed between GluR1 and RAR-α mRNA levels (*r* = −0.091, *p* = 0.863, **D**).

We analyzed the correlations among GluR1, CRH, and RARα mRNA levels in the hypothalamus. In the control group, GluR1 and CRH mRNA levels exhibited a significant negative correlation (*R*^2^ = 0.978, **p < 0.01, Figure 6A), while GluR1 and RARα mRNA levels showed a positive correlation (*R*^2^ = 0.5693, Figure 6B). In contrast, these correlations were absent in the ATRA-treated group, with no significant relationship observed between GluR1 and CRH (*R*^2^ = 0.264, Figure 6C) or between GluR1 and RARα (*R*^2^ = 0.008, Figure 6D). These findings suggest that ATRA treatment alters the regulatory relationship between GluR1, CRH, and RARα at the transcriptional level.

### 3.7 ATRA enhanced dendritic spine density in cultured hypothalamic neurons

**Figure 7:**
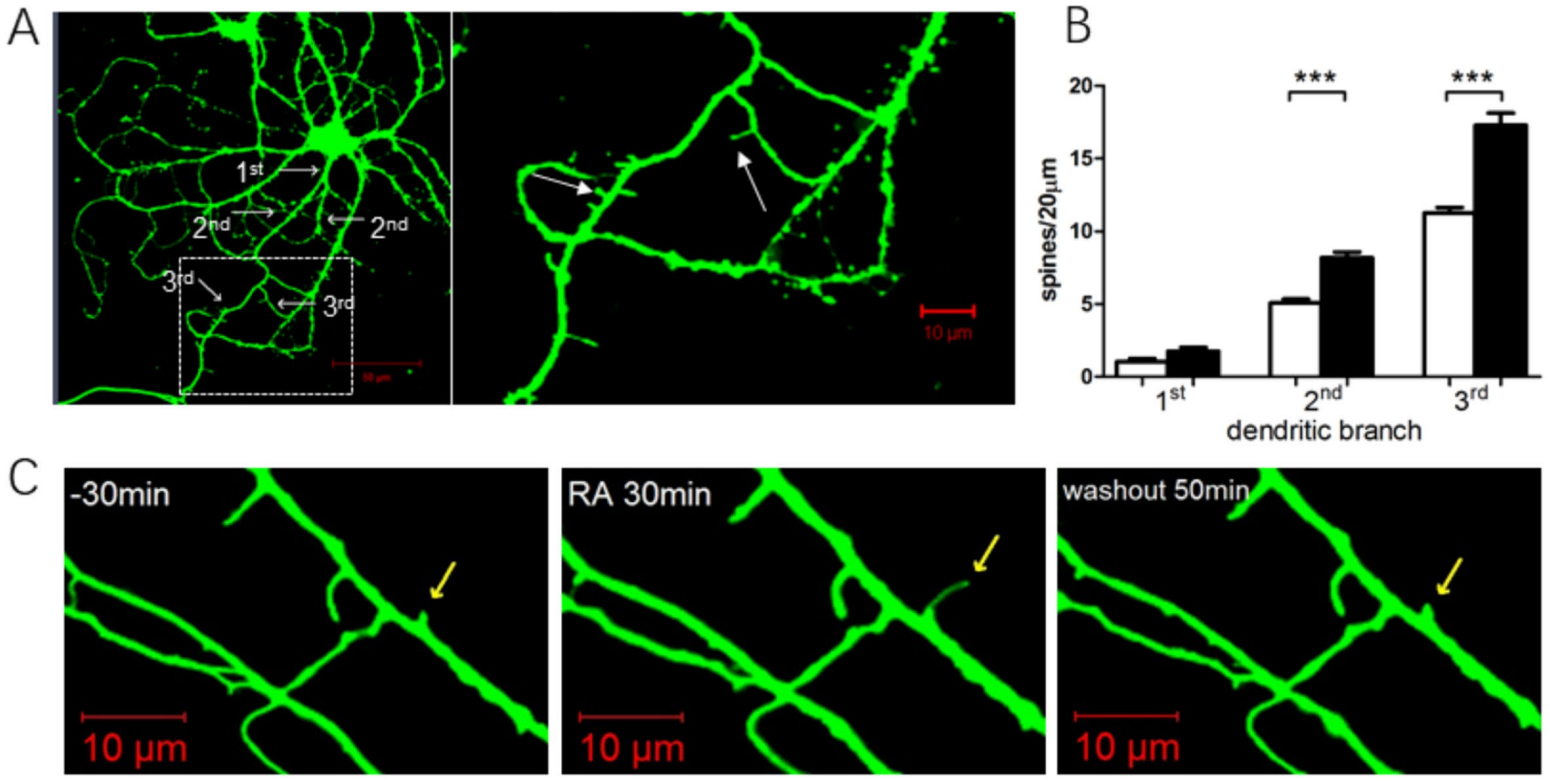
Effects of ATRA administration on dendritic spines. **(A)** Left: Hypothalamic neurons stained using a live-cell dye at day 13, revealing dendritic branching patterns (white arrows). Right: Enlarged view of dendritic spines (white arrows), predominantly thin and mushroom morphologies. **(B)** Quantification of dendritic spine density in primary, secondary, and tertiary dendrites of hypothalamic neurons. Black bars: ATRA group; white bars: DMSO control. (** *p* < 0.0001, *n* = 20 neurons/group). **(C)** Dynamic changes in dendritic spines before, during, and after ATRA treatment. Yellow arrows mark spines undergoing structural modifications. The first 30 min represent baseline before drug application. Data are presented as mean ± SEM; Student’s *t*-tests were used for analysis. Statistical significance between the CON and ATRA groups is denoted by asterisks (***, *p* < 0.01) in the figure.

We analyzed dendritic spine density in 20 randomly selected neurons from both the ATRA-treated and DMSO control groups. Hypothalamic neurons, primarily bipolar (two projections) or tripolar (three projections) with rare multipolar forms, were examined. To ensure consistency, spine density was quantified on primary, secondary, and tertiary dendrites (Figure 7A).

After 24 h of ATRA treatment, hypothalamic neurons exhibited a slight increase in primary dendrite numbers, with significant increases in spine density on secondary and tertiary dendrites. Further analysis revealed that in bipolar neurons, ATRA treatment significantly increased spine density compared to DMSO controls (Figure 7B, *p* < 0.0001). Similarly, in tripolar neurons, ATRA-treated neurons showed significantly higher spine density than controls (Figure 7B, *p* < 0.0001). These findings demonstrate that ATRA promotes dendritic spine formation in hypothalamic neurons, particularly on secondary and tertiary dendrites across both bipolar and tripolar subtypes.

Figure 7C illustrates the dynamic morphological changes in dendritic spines before, during, and after ATRA treatment. Following ATRA application, spines exhibited elongation, which reversed after ATRA washout. Other spines within the field of view showed no significant morphological changes.

## 4 Discussion

In this study, we reported three major findings. First, chronic ATRA treatment induced anxiety- and depression-like behaviors in rats, accompanied by upregulated RARα and CRH expression in the hypothalamus. Second, ATRA-treated rats exhibiting depression-like behaviors showed abnormal expression of AMPA receptor subunits in the hypothalamus. Third, ATRA treatment significantly enhanced dendritic spine density in cultured hypothalamic neurons, with pronounced effects on secondary and tertiary dendrites.

Our study demonstrates that chronic ATRA treatment upregulates hypothalamic RARα and CRH, key regulators of the hypothalamic–pituitary–adrenal (HPA) axis, leading to depression-like behaviors in rats. After 6 weeks of ATRA administration, CRH and RARα mRNA levels increased significantly, correlating with HPA axis hyperactivation. Behavioral changes observed in ATRA-treated rats, such as reduced exploratory activity and increased immobility, align with findings from prior studies linking excessive CRH to HPA axis overactivity and depressive symptoms (Hu et al., 2013; Cai et al., 2010; O’Reilly et al., 2006; Arborelius et al., 1999; Bao et al., 2008; Chen and Napoli, 2008; Qi et al., 2014; Shearer et al., 2010; Wang et al., 2008). Retinoic acid (RA) signaling, mediated by receptors such as RARα, *β*, *γ*, and RXR-γ, directly regulates CRH expression in the hypothalamus (Qi et al., 2014; Ludot et al., 2015). Elevated CRH, a critical HPA axis regulator, is associated with hyperactivity of this system in depression patients (Arborelius et al., 1999; Wang et al., 2008). Notably, RARα expression is increased in the paraventricular nucleus (PVN) of both depressed patients and rodent models, often co-localizing with CRH-expressing neurons (Chen and Napoli, 2008; Ferguson et al., 2005). Long-term ATRA exposure not only activates the HPA axis but also disrupts synaptic plasticity, as evidenced by increased dendritic spine density in hypothalamic neurons (Cai et al., 2010). These structural changes may underlie the behavioral and neuroendocrine alterations observed in depression. Studies further confirm that RA administration enhances depression-like behaviors and HPA axis activation (Hu et al., 2013; O’Reilly et al., 2006). Collectively, these findings highlight ATRA’s role in hypothalamic dysfunction and depression, suggesting that RA signaling pathways may represent therapeutic targets for mood disorders. Future research should explore how modulating RARα activity might normalize HPA axis function and alleviate depressive symptoms.

Our study revealed that chronic ATRA treatment in rats exhibiting depression-like behaviors is associated with significant alterations in AMPA receptor subunit expression. Specifically, we observed increased mRNA levels of GluR1 and GluR2, alongside elevated GluR1 protein levels, and a decrease in GluR4 expression. These findings underscore the critical role of AMPA receptors in synaptic efficacy and excitatory neurotransmission within the central nervous system (Palmer et al., 2004). AMPA receptor subunit composition directly influences channel function and trafficking, with dysregulation implicated in depression (Duric et al., 2013; Gibbons et al., 2012). Elevated glutamate concentrations, as seen in pathological states, can impair synaptic plasticity and induce excitotoxicity (Duric et al., 2013; Rosa et al., 2002). ATRA, acting via RARα, increases synaptic GluR1 levels, a mechanism supported by our observation of co-localization between RARα and GluR1 in CRH-expressing neurons. Interestingly, while a negative correlation between GluR1 and CRH mRNA levels was noted in controls, this relationship was disrupted by chronic ATRA treatment. Similarly, the positive correlation between GluR1 and RARα mRNA levels in controls was absent in ATRA-treated rats. These disruptions suggest that ATRA alters the regulatory dynamics among these molecules. Previous studies have linked elevated GluR1 and GluR2 levels to stress and fear memory consolidation (Shimizu et al., 2016; Thoeringer et al., 2012). Additionally, GluR4, a key determinant of synaptic AMPA receptors, is associated with major depression and other neuropsychiatric disorders (Beneyto and Meador-Woodruff, 2006; Pickard et al., 2006). Our results indicate that hypothalamic glutamate receptor dysregulation in ATRA-treated rats may contribute to disrupted synaptic connections, aligning with theories that link impaired synaptic plasticity to depression (Rial et al., 2015). These findings highlight the potential role of AMPA receptor modulation in the pathophysiology of depression.

Synapsin I and PSD-95 are critical for synapse formation and function, with their dysregulation implicated in depression (Holmes et al., 2019). While some studies report reduced synapsin I in stress models (Feyissa et al., 2009; Marrocco et al., 2012) and elevated levels in chronically stressed rats (Bessa et al., 2013), our study found no significant changes in synapsin I protein levels. However, we observed a decrease in PSD-95 protein levels in ATRA-treated rats, accompanied by increased GluR1 levels. PSD-95, essential for anchoring AMPA receptors and signaling proteins, may alter synaptic function when its levels change (Kim and Sheng, 2004). Consistent with our findings, reduced PSD-95 has been reported in the anterior prefrontal cortex of depressed patients (Feyissa et al., 2009) and following CRH exposure (Andres et al., 2013), though increased levels in the amygdala of depressed patients (Karolewicz et al., 2009) highlight region-specific effects.

Our findings show that ATRA treatment significantly increases dendritic spine density in hypothalamic neurons, particularly in secondary and tertiary dendrites, highlighting its role in modulating synaptic structure and plasticity. This structural change may reflect enhanced synaptic connectivity, potentially altering neuronal excitability and circuit function. Previous studies have linked ATRA to synaptic plasticity in other brain regions, such as the hippocampus, where it influences dendritic spine morphology (Cai et al., 2010; Shearer et al., 2010). The selective enhancement in secondary and tertiary dendrites suggests region-specific effects, possibly due to functional specialization. These changes may have implications for understanding depression pathophysiology, as reduced synaptic density and plasticity are associated with depressive states (Rial et al., 2015). Given that the effect of ATRA is both dose-dependent and time-dependent, variations in treatment duration may result in differing levels of pathway activation, thereby leading to relatively inconsistent outcomes (Qiao et al., 2016). While ATRA-induced spine increases might seem contradictory, they could represent a compensatory mechanism or contribute to maladaptive circuit changes, such as heightened stress responsiveness. Future research should explore how these structural changes influence synaptic function and neurotransmission, particularly in relation to HPA axis regulation, to clarify the therapeutic potential of targeting ATRA signaling pathways.

Our results suggest that ATRA-induced depression-like behaviors are associated with hypothalamic glutamate signaling abnormalities, including elevated glutamate concentrations and altered AMPA receptor subunit expression. After 6 weeks of ATRA treatment, glutamate levels in the hypothalamus were significantly higher, paralleling behavioral changes. This aligns with the hyperactivity of the HPA axis and overactive glutamatergic system observed in depression (Gao and Bao, 2011). Glutamate’s role in HPA axis regulation, particularly through PVN innervation, may contribute to HPA axis sensitization under chronic stress (Evanson and Herman, 2015). Discrepancies in PSD-95 findings across studies may stem from regional differences and variations in experimental methodologies or participant characteristics (Kristiansen and Meador-Woodruff, 2005; Toro and Deakin, 2005). Future research should focus on identifying specific AMPA receptor subunits involved in HPA axis regulation and their therapeutic potential in depression.

## Data Availability

The raw data supporting the conclusions of this article will be made available by the authors, without undue reservation.
